# Vaccinatiebereidheid en opleidingsniveau

**DOI:** 10.1007/s12508-021-00317-8

**Published:** 2021-10-20

**Authors:** Sarah Vader, Ellen Uiters, Fons van der Lucht, Carolien Smits, Floor Kroese, Marijn de Bruin

**Affiliations:** 1 Rijksinstituut voor Volksgezondheid en Milieu, Bilthoven, Nederland; 2 Pharos Expertisecentrum Gezondheidsverschillen, Utrecht, Nederland

**Keywords:** vaccinatiebereidheid, COVID-19, opleidingsniveau, Vaccination willingness, COVID-19, Educational level

## Abstract

Vaccinatie is een belangrijk onderdeel in de bestrijding van het COVID-19-virus. Een voorspeller van het aandeel mensen dat daadwerkelijk een vaccinatie zal nemen is de vaccinatiebereidheid onder de bevolking. Uit buitenlandse literatuur blijkt dat de vaccinatiebereidheid onder mensen met een lagere sociaaleconomische status lager ligt dan onder andere groepen. In deze bijdrage beschrijven we in hoeverre dit ook in Nederland het geval is en laten we zien hoe risicoperceptie, vertrouwen in de werking en veiligheid van het vaccin en gezondheidsvaardigheden hier mogelijk mee samenhangen. Tot slot belichten we een aantal interventiestrategieën die positief aan de vaccinatiebereidheid onder laagopgeleiden kunnen bijdragen*.*

## Abstract (en)

Vaccination is an important part of the fight against COVID-19 virus. A predictor of the proportion of people who will actually take a vaccination is the willingness to vaccinate among the population. Literature shows that vaccination willingness among people with lower socioeconomic status is lower than among other groups. In this contribution we describe to what extent this is also the case in the Netherlands and show how risk perception, trust in the effects and safety of the vaccine, and health literacy may be related to this. Finally, we highlight a number of intervention strategies that can make a positive contribution to vaccination willingness among lower educated groups.

## Inleiding

Sinds begin 2021 is vaccinatie een centraal onderdeel in de bestrijding van het COVID-19-virus. Om in te kunnen schatten hoeveel mensen zich willen laten vaccineren wordt gekeken naar de vaccinatiebereidheid. De vraag is hierbij in hoeverre deze bereidheid voor de verschillende subgroepen in de bevolking even hoog is. Buitenlandse onderzoeken laten zien dat mensen uit lagere sociaaleconomische groepen over het algemeen minder bereid zijn zich te laten vaccineren dan mensen uit hogere sociaaleconomische groepen. In onderzoeken in Groot-Brittannië en de VS kwam bijvoorbeeld naar voren dat een laag inkomen samenhangt met negatieve opvattingen over het COVID-19-vaccin en dat de vaccinatiebereidheid toeneemt naarmate het opleidingsniveau stijgt [1, 2]. In deze bijdrage beschrijven we in hoeverre dit beeld voor COVID-19-vaccinatie ook in het begin van de vaccinatiecampagne in Nederland zichtbaar is en gaan we in op belangrijke factoren die samenhangen met de vaccinatiebereidheid. Daarnaast beschrijven we enkele interventiestrategieën die positief aan de vaccinatiebereidheid kunnen bijdragen, zoals het inzetten van sleutelfiguren en het slechten van praktische barrières.

## Corona Gedragsunit

De WHO geeft gedragswetenschappen een belangrijke plaats in de bestrijding van pandemieën, naast andere essentiële expertise, zoals virologie, epidemiologie en geneeskunde. In lijn daarmee werd in het voorjaar van 2020, tijdens de eerste golf van de COVID-19-pandemie, de Corona Gedragsunit opgericht, met als doel om wetenschappelijke kennis en expertise over gedrag op gecoördineerde wijze in te kunnen zetten bij de bestrijding van de coronapandemie [3]. Naast literatuuronderzoek, interviews en het in kaart brengen van voorbeelden uit de praktijk vormt periodiek vragenlijstonderzoek een belangrijke pijler van de gegevensverzameling. Het doel van dit vragenlijstonderzoek is om inzicht te krijgen in de belangrijkste gedragingen die cruciaal zijn bij een succesvolle bestrijding van de pandemie. Ook vaccinatiebereidheid is een onderwerp dat aandacht krijgt in het vragenlijstonderzoek.

## Vaccinatiebereidheid

Voor deze bijdrage hebben we de tiende ronde van het vragenlijstonderzoek gebruikt. Tussen 10 en 14 februari namen 54.363 mensen in de leeftijd vanaf zestien jaar deel. Van hen was 36% man en 64% vrouw. In het vragenlijstonderzoek is een onderscheid gemaakt tussen laag- (geen, basis, lbo, vmbo), middelbaar (havo/vwo, mbo) en hoogopgeleiden (hbo, wo).

Met de vraag ‘Als u uitgenodigd wordt voor een vaccinatie tegen het coronavirus, wilt u zich dan laten vaccineren?’ werd de vaccinatiebereidheid onder de deelnemers uitgevraagd. In totaal gaf 84% van de algehele bevolking aan bereid te zijn zich te laten vaccineren. Tabel 1 laat zien dat dit percentage onder laag- en middelbaar opgeleiden lager is dan onder hoogopgeleiden. Dit werd gevonden voor alle leeftijdsgroepen.

**Table 1:** 

	laag opgeleid (*n* = 6.368)	middelbaar opgeleid (*n* = 15.043)	hoog opgeleid (*n* = 32.309)
*ja*	82%	81%	87%
*nee*	6%	7%	4%
*weet ik niet*	12%	12%	9%

Voor dit onderzoek zijn de gegevens uit het vragenlijstonderzoek van de Corona Gedragsunit gebruikt. Kijk voor gegevens van het trendonderzoek naar vaccinatiebereidheid en opleidingsniveau op https://coronadashboard.rijksoverheid.nl/landelijk/vaccinaties.

De vaccinatiebereidheid hangt binnen de opleidingsgroepen ook samen met leeftijd. Naarmate de leeftijd stijgt, wordt het verschil in vaccinatiebereidheid tussen opleidingsgroepen kleiner. Zo is in de leeftijdsgroep 25–39 jaar het verschil in vaccinatiebereidheid tussen laag- en hoogopgeleiden 23 procentpunt. Voor de groep 70-plussers is dit gedaald naar 5%.

## Risicoperceptie

Uit gedragswetenschappelijk onderzoek weten we dat risicoperceptie een belangrijke factor is wat betreft de vaccinatiebereidheid [4]. Naarmate mensen de gevolgen van een besmetting ernstiger inschatten, zijn ze eerder bereid zich te laten vaccineren. Met betrekking tot COVID-19 blijkt op basis van de cijfers van het vragenlijstonderzoek dat de veronderstelde ernst van COVID-19 onder laagopgeleiden hoger is dan onder de andere opleidingsgroepen. Van de laagopgeleiden zou 71% het heel erg vinden om met het coronavirus besmet te raken. Voor middelbaar en hoogopgeleiden ligt dit percentage lager (respectievelijk 58% en 55%). Het percentage laagopgeleiden dat de kans groot acht om de komende tijd met het virus besmet te raken is 8%. Bij middelbaar opgeleiden is dit 14% en bij hoogopgeleiden 12%. Net als bij vaccinatiebereidheid hangt de risicoperceptie in alle opleidingsgroepen samen met leeftijd. Naarmate respondenten jonger zijn, daalt ook de angst om besmet te raken met het COVID-19-virus en ernstig ziek te worden. Ook als rekening wordt gehouden met verschillen in risicoperceptie en leeftijd, geslacht en gezondheid, blijven verschillen in vaccinatiebereidheid tussen opleidingsgroepen zichtbaar. De kans dat mensen twijfelen of aangeven geen vaccin te willen is ook na correctie voor deze verschillen hoger onder laag- en middelbaar opgeleide groepen dan onder hoogopgeleiden.

## Vertrouwen

Naast risicopercepties zijn ook vertrouwen in de werking van het vaccin en vertrouwen in de overheid belangrijke factoren die een rol spelen bij de vaccinatiebereidheid [5]. Als er geen vertrouwen in de werking en effectiviteit van het vaccin is, zijn mensen minder bereid zich te laten vaccineren [5]. Ook wanneer mensen geen vertrouwen hebben in de overheid, is hun bereidheid zich te laten vaccineren kleiner [6, 7]. Zo laat Nederlands onderzoek zien dat mensen met meer vertrouwen in de overheid ook een hogere vaccinatiebereidheid hebben [6]. Wantrouwen in de overheid en minder goede ervaringen met de overheid worden zowel in Nederland als in het buitenland meer gevonden in laagopgeleide groepen, dan in andere opleidingsgroepen [8]. Cijfers van het vragenlijstonderzoek laten zien dat vertrouwen in de bescherming van het vaccin tegen het coronavirus lager is onder laag- (52%) en middelbaar opgeleiden (56%), dan onder hoogopgeleiden (71%). De angst voor bijwerkingen van het vaccin is onder laag- (26%) en middelbaar opgeleiden (25%) hoger dan onder hoogopgeleiden (15%). Vertrouwen in de aanpak van de overheid was daarentegen voor alle groepen vrijwel gelijk (laag 40%, middelbaar 40% en hoog 42%). Wanneer in verdiepende analyses rekening wordt gehouden met verschillen in vertrouwen in de aanpak van de Nederlandse overheid, leeftijd, geslacht en gezondheid, blijven verschillen in vaccinatiebereidheid tussen opleidingsgroepen zichtbaar. De kans dat mensen twijfelen of aangeven geen vaccin te willen is hoger onder laag- en middelbaar opgeleide groepen dan onder de hoogopgeleiden. Dit beeld verandert als vervolgens ook nog rekening wordt gehouden met vertrouwen in de werking en veiligheid van het vaccin. Dit suggereert dat het verband tussen opleidingsniveau en vaccinatiebereidheid grotendeels wordt verklaard door verschillen in vertrouwen in de werking en veiligheid van het vaccin.

## Informatiebronnen

Om mensen in staat te stellen zelf een weloverwogen keuze te maken om zich wel of niet tegen COVID-19 te laten vaccineren, is het belangrijk dat mensen gebruik kunnen maken van betrouwbare en begrijpelijke informatie (zie kader 1).

### Kader 1 Gezondheidsvaardigheden

Gezondheidsvaardigheden helpen om gezond te blijven en om optimaal gebruik te maken van het aanbod van de gezondheidszorg, zoals een COVID-19-vaccin. Mensen met beperkte gezondheidsvaardigheden hebben moeite met het vinden, begrijpen, op waarde schatten en toepassen van gezondheidsgerelateerde informatie bij het nemen van beslissingen over gezondheid. Drie van de tien Nederlanders hebben beperkte gezondheidsvaardigheden [9]. Ouderen, laagopgeleiden en niet-westerse migranten zijn oververtegenwoordigd in de groep van mensen met beperkte gezondheidsvaardigheden. De Nederlandse vaccinatiestrategie gaat ervan uit dat mensen ófwel een groot vertrouwen hebben in autoriteiten, zoals de overheid of de huisarts, ófwel over voldoende gezondheidsvaardigheden beschikken. Mensen moeten als eerste stap naar vaccinatiebereidheid begrijpelijke vaccinatiegerelateerde informatie kunnen vinden. De hoeveelheid informatie lijkt hierbij niet het probleem, wel het vinden van begrijpelijke informatie. Daarbij is het goed om te weten dat een op de negen Nederlanders moeite heeft met lezen en schrijven. Voor migranten geldt dat ze de Nederlandse taal vaak niet op voldoende niveau beheersen, terwijl veel officiële informatie op een relatief hoog taalniveau en in het Nederlands wordt aangeboden. Informatie over corona verandert bovendien voortdurend en wordt vanuit vele bronnen gepresenteerd. Die bronnen spreken elkaar soms tegen. Migranten gebruiken daarbij vaak informatie uit het land van herkomst of uit landen waar familieleden wonen. Ook die informatie strookt niet altijd met de officiële RIVM-informatie. Bij de tweede stap moeten mensen al die informatie op waarde schatten. Welke bron is het meest betrouwbaar? Als derde stap moeten mensen de informatie over vaccinatie vertalen naar de eigen situatie. Daarbij moeten ze voor zichzelf op basis van informatie over vaccinatie beslissen en voor- en nadelen afwegen. Veel mensen toetsen hun afwegingen aan mensen die ze vertrouwen. Dat kunnen familieleden zijn, vrienden, collega’s, sleutelpersonen in de wijk of vrijwilligers, of sociale of zorgprofessionals. De Rijksoverheidsstrategie kan dan ook aan doelmatigheid winnen door begrijpelijke informatie aan te bieden via kanalen die gebruikt worden door de mensen om wie het gaat. Verschillende GGD’en en Pharos werken aan begrijpelijk materiaal om informatie over het vaccin toegankelijk en begrijpelijk voor iedereen te maken, zoals informatieve filmpjes.

Vaccin tegen Corona – Pharos (https://www.pharos.nl/coronavirus/coronavaccinatie/).

## Interventiestrategieën

De kleinere vaccinatiebereidheid onder groepen met een lage opleiding vraagt om specifieke aandacht voor de zorgen en informatiebehoeften bij deze groepen. Daarbij zijn begrijpelijke informatie en vertrouwen in degenen die informatie over het vaccin verstrekken van belang. Het inzetten van sleutelfiguren en zorgverleners uit lokale gemeenschappen bij het verstrekken van COVID-gerelateerde informatie kan het vertrouwen in de werking en de veiligheid van COVID-19-vaccinaties bevorderen [10]. Ook is het van belang aandacht te schenken aan de groep twijfelaars en in te gaan op hun specifieke vragen en behoeften.

Uit bestaande inzichten van de Corona Gedragsunit blijkt dat ook praktische barrières een belangrijke rol spelen bij het achterblijven van vaccinatiedeelname onder lagere sociaaleconomische groepen. Door het slechten van deze barrières, zoals de bereikbaarheid en toegankelijkheid van vaccinatielocaties, wordt het makkelijker om een vaccinatie te gaan halen, wat positief kan bijdragen aan de vaccinatiebereidheid. Het aanbod kan praktisch toegankelijker worden gemaakt door de vaccinatielocaties dicht bij de plek waar mensen wonen te plannen, en door het mogelijk te maken om zonder afspraak binnen te lopen.

Meer informatie over de ontwikkeling en inzet van interventies rond vaccinatiebereidheid is te vinden via de Corona Gedragsunit (https://www.rivm.nl/documenten/verkenning-factoren-van-invloed-op-deelname-aan-covid-19-vaccinatie).

## References

[1] Paul E, Steptoe A, Fancourt D (2021). Attitudes towards vaccines and intention to vaccinate against COVID-19: Implications for public health communications. Lancet Reg Health Eur.

[2] Malik AA, McFadden SM, Elharake J, Omer SB (2020). Determinants of COVID-19 vaccine acceptance in the US. EClinicalMedicine.

[3] Corona Gedragsunit RIVM. 10e ronde vragenlijstenonderzoek. 2021. https://www.rivm.nl/gedragsonderzoek/maatregelen-welbevinden/resultaten-10e-ronde-gedragsonderzoek/vaccinatiebereidheid. Geraadpleegd op: 28 jul 2021.

[4] van Lier A, van de Kassteele J, de Hoogh P, Drijfhout P, de Melker H (2014). Vaccine uptake determinants in the Netherlands. Eur J Public Health.

[5] Mouter N, de Ruijter A, Kessels R (2020). De meeste Nederlanders staan niet vooraan in de rij voor een COVID-19 vaccin. Beleidsrapport over de hoofdresultaten van een keuze experiment naar de voorkeuren van Nederlanders voor een COVID-19 vaccin.

[6] Onderzoeksrapport Ipsos/NOS. Nederlanders over de coronavaccinatie. 2021. https://www.ipsos.com/sites/default/files/ct/news/documents/2021-01/ipsos_nos_vaccinatiebereidheid_v3.0.pdf. Geraadpleegd op: 28 jul 2021.

[7] Greenberg J, Dubé E, Driedger M (2017). Vaccine hesitancy: in search of the risk communication comfort zone. PLoS Curr.

[8] Edwards B, Biddle N, Gray M, Sollis K (2021). COVID-19 vaccine hesitancy and resistance: correlates in a nationally representative longitudinal survey of the Australian population. PLoS ONE.

[9] Heijmans M, Braber A, Rademakers J (2019). Hoe gezondheidsvaardig is Nederland? Factsheet gezondheidsvaardigheden – Cijfers 2019.

[10] Razai M, Osama T, McKechnie D (2021). COVID-19 vaccine hesitancy among ethnic minority groups. BMJ.

